# Aqua­bis­(2,2′-bi­pyridine-κ^2^*N*,*N*′)(isonicotinamide-κ*N*)ruthenium(II) bis­(trifluoromethanesulfonate)

**DOI:** 10.1107/S2414314624001147

**Published:** 2024-02-08

**Authors:** Liam Faubert, Hadi D. Arman, Rafael A. Adrian

**Affiliations:** a Department of Chemistry and Biochemistry, University of the Incarnate Word, San Antonio, Texas 78209, USA; b Department of Chemistry The University of Texas at San Antonio,San Antonio Texas 78249 USA; Goethe-Universität Frankfurt, Germany

**Keywords:** crystal structure, ruthenium(II), octa­hedral geometry, 2,2′-bi­pyridine, bidentate ligand, isonicotinamide, hydrogen bonding, π–π stacking

## Abstract

In the title complex, [Ru(C_10_H_8_N_2_)_2_(C_6_H_6_N_2_O)(H_2_O)](CF_3_SO_3_)_2_, the central Ru^II^ atom is sixfold coordinated by two bidentate 2,2′-bi­pyridine, an isonic­otinamide ligand, and a water mol­ecule in a distorted octa­hedral environment with tri­fluoro­methane­sulfonate ions completing the outer coordination sphere of the complex. Hydrogen bonding involving the water mol­ecule and weak π–π stacking inter­actions between the pyridyl rings in adjacent mol­ecules contribute to the alignment of the complexes in columns parallel to the *c* axis.

## Abstract 2

In the crystal structure of the title compound, the ruthenium(II) metal center is surrounded by two bidentate 2,2′-bi­pyridine, an isonicotinamide ligand, and a water in a distorted octa­hedral geometry. Hydrogen bonding is present between counter-ion and ligand.

## Graphical abstract

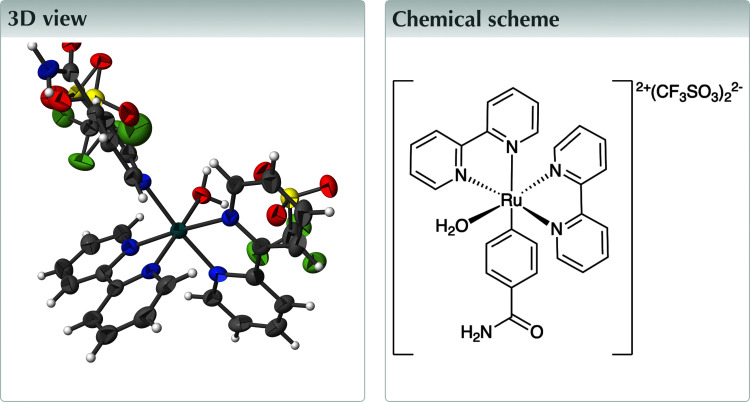

## Structure description

**Figure 1:**
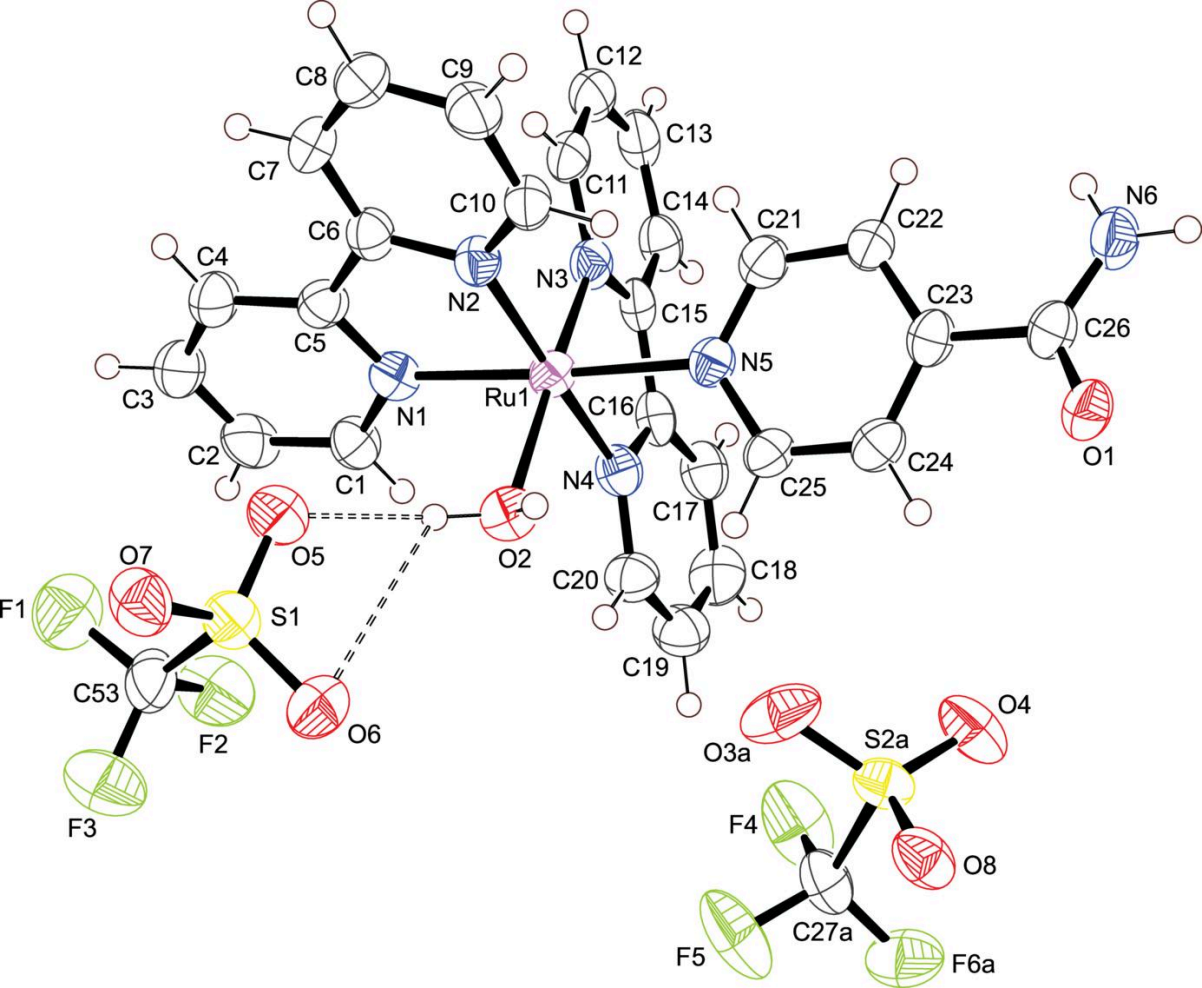
Asymmetric unit of the title compound with displacement ellipsoids drawn at the 50% probability level; hydrogen bonds shown as dashed lines. The minor occupied sites of the disordered tri­fluoro­methane­sulfonate ion are omitted for clarity.

Over the last three years, a lot of effort has been directed into studying ruthenium(II) bis­(bi­pyridine) complexes due to their catalytic properties (Griffin *et al.*, 2021 ▸), luminescence (Cuéllar *et al.*, 2021 ▸) and biological activity (Al–Wahaib *et al.*, 2021 ▸; Allison *et al.*, 2021 ▸). Last year, bi­pyridine ruthenium(II) complexes with halogen-substituted salicylates showed promising *in vitro* anti­proliferative activity against MCF-7 (breast cancer) and U-118 MG (human glioma) cell lines (Schoeller *et al.*, 2023 ▸). Our research group’s inter­est currently lies in synthesizing metal complexes with application as anti­proliferative agents; as part of our research in this area, we describe the synthesis and structure of the title ruthenium(II) complex, Fig. 1 ▸.

The asymmetric unit contains the title compound, with four symmetry-related entities inside the unit cell. The ruthenium(II) ion shows a distorted octa­hedral coordination environment defined by two 2,2′-bi­pyridine ligands, one isonicotinamide ligand and a water mol­ecule. Tri­fluoromethane­sulfonate ions sit in the outer coord­ination sphere, balancing the charge of the metal complex. All the Ru—N bond bond lengths are in good agreement with comparable ruthenium(II) bis­(2,2′-bi­pyridine) complexes currently available in the Cambridge Structural Database (CSD, version 5.45, Nov 2023; Groom *et al.*, 2016 ▸; Huang & Ogawa, 2006 ▸, refcode LECFOP; Keniley Jr *et al.*, 2013 ▸, refcode AROJIB01; Yoshikawa *et al.*, 2016 ▸, refcode DACBER; de Souza *et al.*, 2017 ▸, refcode TAWQOA), The N—Ru—N angles found in the bi­pyridine ligands also concur with the values reported in the previously referenced ruthenium(II) bi­pyridine complexes. The Ru—O bond length matches well with the distance in other ruthenium complexes with a coordinating water mol­ecule (Gupta *et al.*, 1992 ▸, refcode KUPBUS; Bonnet *et al.*, 2003 ▸, refcode IPESIF; Ghaderian *et al.*, 2020 ▸, refcode LAGCIJ). All relevant bonds and angles are presented in Table 1 ▸.

**Figure 2:**
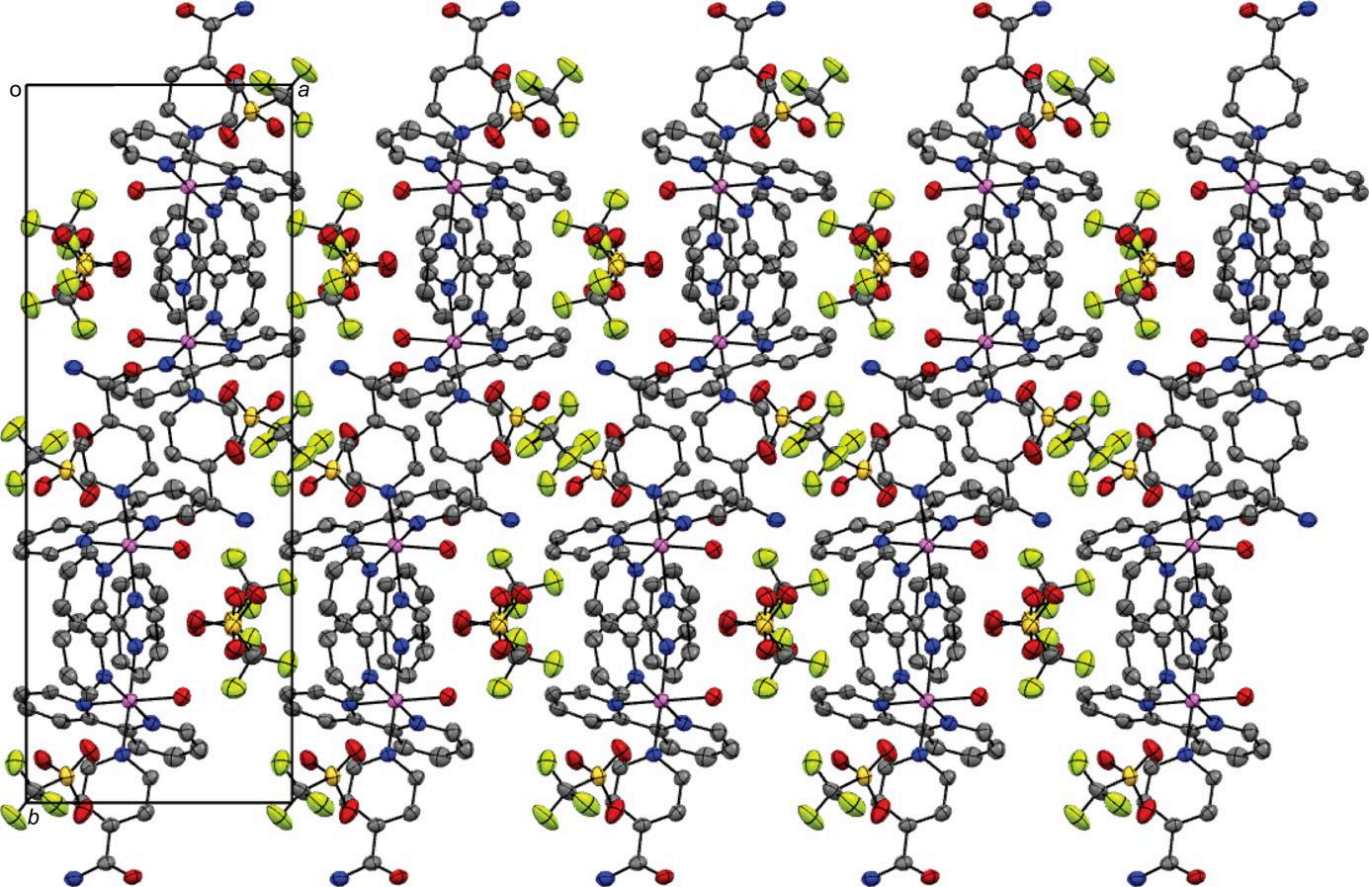
Perspective view of the crystal packing of the title complex parallel to the *c* axis; H atoms and the minor occupied sites of the disordered tri­fluoro­methane­sulfonate ion are omitted for clarity.

The packing diagram reveals the stacking of the asymmetric units in columns observable when looking at the crystal parallel to the *c*axis (Fig. 2 ▸). Contiguous pyridine rings show weak π–π stacking inter­actions, with a centroid-to-centroid distance (*Cg*⋯*Cg*) of 3.791 (4) Å and an offset distance of 1.559 Å, which are responsible for the stacking into columns. Tri­fluoro­methane­sulfonate ions sit in the gap between columns and are held in place by hydrogen bonds between one of the oxygen atoms of the tri­fluoro­methane­sulfonate ion and one of the hydrogen atoms in the water mol­ecule, as well as between the oxygen in the isonicotinamide ligand and the second hydrogen in the water mol­ecule (Table 2 ▸).

## Synthesis and crystallization

The title compound was synthesized by the reaction of *cis*-Ru(bpy)_2_Cl_2_ (0.100 g, 0.206 mmol) with Ag(CF_3_SO_3_) (0.106 g, 0.412 mmol) in 50 ml of EtOH. After heating and stirring for about 1 h at 323.15 K, the solution was filtrated using a PTFE syringe filter to remove AgCl. Isonicotinamide (0.050 g, 0.41 mmol) was then added to the mixture and the resulting solution was heated at 338.15 K until the volume was reduced to about 10 ml. Crystal suitable for X-ray diffraction were grown by vapor diffusion of diethyl ether over a saturated aceto­nitrile solution of the title complex.

## Refinement

Crystal data, data collection and structure refinement details are summarized in Table 3 ▸. One of the tri­fluoro­methane­sulfonate ions has four atoms disordered over two sets of sites. The site occupation factors of the disordered atoms were set to 0.85 and 0.15, respectively. The displacement parameters of the minor occupied sites were restrained to an isotropic behavior. The C—S distance of the minor occupied sites was restrained to 1.70 (2) Å. H atoms were refined using a riding model with C—H = 0.95 Å and *U*_iso_(H) = 1.2*U*_eq_(C) or O—H = 0.89 Å and *U*_iso_(H) = 1.5*U*_eq_(O).

## Supplementary Material

Crystal structure: contains datablock(s) I. DOI: 10.1107/S2414314624001147/bt4147sup1.cif

Structure factors: contains datablock(s) I. DOI: 10.1107/S2414314624001147/bt4147Isup2.hkl

Supporting information file. DOI: 10.1107/S2414314624001147/bt4147Isup4.mol

CCDC reference: 2330544

Additional supporting information:  crystallographic information; 3D view; checkCIF report

## Figures and Tables

**Table 1:** Selected geometric parameters (Å, °)

Ru1—N3	2.030 (6)	Ru1—N2	2.069 (5)
Ru1—N1	2.047 (6)	Ru1—N5	2.087 (5)
Ru1—N4	2.050 (6)	Ru1—O2	2.145 (5)
			
N3—Ru1—N1	91.1 (2)	N4—Ru1—N5	86.8 (2)
N3—Ru1—N4	79.4 (2)	N2—Ru1—N5	95.4 (2)
N1—Ru1—N4	99.3 (2)	N3—Ru1—O2	171.7 (2)
N3—Ru1—N2	96.3 (2)	N1—Ru1—O2	86.9 (2)
N1—Ru1—N2	78.6 (2)	N4—Ru1—O2	93.0 (2)
N4—Ru1—N2	175.2 (2)	N2—Ru1—O2	91.2 (2)
N3—Ru1—N5	90.7 (2)	N5—Ru1—O2	92.1 (2)
N1—Ru1—N5	173.9 (2)		

**Table 2:** Hydrogen-bond geometry (Å, °)

*D*—H⋯*A*	*D*—H	H⋯*A*	*D*⋯*A*	*D*—H⋯*A*
O2—H2*A*⋯O1^i^	0.89	1.73	2.617 (7)	174
O2—H2*B*⋯O5	0.89	1.90	2.775 (8)	169
N6—H6*A*⋯O6^i^	0.88	2.32	3.190 (9)	169

Symmetry code: (i) 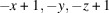.

**Table 3:** Experimental details

Crystal data	
Chemical formula	[Ru(C_10_H_8_N_2_)_2_(C_6_H_6_N_2_O(H_2_O)](CF_3_SO_3_)_2_
*M*_r_	851.72
Crystal system, space group	Monoclinic, *P*2_1_/*c*
Temperature (K)	100
*a*, *b*, *c* (Å)	9.7878 (1), 26.2103 (3), 12.6443 (1)
β (°)	97.202 (1)
*V* (Å^3^)	3218.19 (6)
*Z*	4
Radiation type	Cu *K*α
μ (mm^−1^)	6.02
Crystal size (mm)	0.11 × 0.06 × 0.04

Data collection	
Diffractometer	XtaLAB Synergy, Dualflex, HyPix
Absorption correction	Gaussian (*CrysAlis PRO*; Rigaku OD, 2023 ▸)
*T*_min_, *T*_max_	0.099, 0.513
No. of measured, independent and observed [*I* > 2σ(*I*)] reflections	35289, 5777, 5536
*R*_int_	0.072
(sin θ/λ)_max_ (Å^−1^)	0.599

Refinement	
*R*[*F*^2^ > 2σ(*F*^2^)], *wR*(*F*^2^), *S*	0.071, 0.159, 1.06
No. of reflections	5777
No. of parameters	496
No. of restraints	25
H-atom treatment	H-atom parameters constrained
Δρ_max_, Δρ_min_ (e Å^−3^)	1.34, −1.09

Computer programs: *CrysAlis PRO* (Rigaku OD, 2023 ▸), *SHELXT2018/2* (Sheldrick, 2015*a* ▸), *SHELXL2018/3* (Sheldrick, 2015*b* ▸), and *OLEX2* (Dolomanov *et al.*, 2009 ▸).

## full crystallographic data

### Aquabis(2,2'-bipyridine-κ2N,N')(isonicotinamide-κN)ruthenium(II) bis(trifluoromethanesulfonate) Crystal data

**Table:**

[Ru(C_10_H_8_N_2_)_2_(C_6_H_6_N_2_O)(H_2_O)](CF_3_O_3_S)_2_	*F*(000) = 1712
*M**_r_* = 851.72	*D*_x_ = 1.758 Mg m^−^^3^
Monoclinic, *P*2_1_/*c*	Cu *K*α radiation, λ = 1.54184 Å
*a* = 9.7878 (1) Å	Cell parameters from 24511 reflections
*b* = 26.2103 (3) Å	θ = 3.4–75.8°
*c* = 12.6443 (1) Å	µ = 6.02 mm^−^^1^
β = 97.202 (1)°	*T* = 100 K
*V* = 3218.19 (6) Å^3^	Plate, clear dark red
*Z* = 4	0.11 × 0.06 × 0.04 mm

### Aquabis(2,2'-bipyridine-κ2N,N')(isonicotinamide-κN)ruthenium(II) bis(trifluoromethanesulfonate) Data collection

**Table:**

XtaLAB Synergy, Dualflex, HyPix diffractometer	5777 independent reflections
Radiation source: micro-focus sealed X-ray tube, PhotonJet (Cu) X-ray Source	5536 reflections with *I* > 2σ(*I*)
Mirror monochromator	*R*_int_ = 0.072
Detector resolution: 10.0000 pixels mm^-1^	θ_max_ = 67.5°, θ_min_ = 3.9°
ω scans	*h* = −11→10
Absorption correction: gaussian (CrysAlisPro; Rigaku OD, 2023)	*k* = −26→31
*T*_min_ = 0.099, *T*_max_ = 0.513	*l* = −15→15
35289 measured reflections	

### Aquabis(2,2'-bipyridine-κ2N,N')(isonicotinamide-κN)ruthenium(II) bis(trifluoromethanesulfonate) Refinement

**Table:**

Refinement on *F*^2^	Primary atom site location: dual
Least-squares matrix: full	Hydrogen site location: mixed
*R*[*F*^2^ > 2σ(*F*^2^)] = 0.071	H-atom parameters constrained
*wR*(*F*^2^) = 0.159	*w* = 1/[σ^2^(*F*_o_^2^) + (0.010*P*)^2^ + 35.*P*] where *P* = (*F*_o_^2^ + 2*F*_c_^2^)/3
*S* = 1.06	(Δ/σ)_max_ < 0.001
5777 reflections	Δρ_max_ = 1.34 e Å^−^^3^
496 parameters	Δρ_min_ = −1.09 e Å^−^^3^
25 restraints	

### Aquabis(2,2'-bipyridine-κ2N,N')(isonicotinamide-κN)ruthenium(II) bis(trifluoromethanesulfonate) Special details

**Table:**

Geometry. All esds (except the esd in the dihedral angle between two l.s. planes) are estimated using the full covariance matrix. The cell esds are taken into account individually in the estimation of esds in distances, angles and torsion angles; correlations between esds in cell parameters are only used when they are defined by crystal symmetry. An approximate (isotropic) treatment of cell esds is used for estimating esds involving l.s. planes.

### Aquabis(2,2'-bipyridine-κ2N,N')(isonicotinamide-κN)ruthenium(II) bis(trifluoromethanesulfonate) Fractional atomic coordinates and isotropic or equivalent isotropic displacement parameters (Å^2^)

**Table:**

	*x*	*y*	*z*	*U*_iso_*/*U*_eq_	Occ. (<1)
Ru1	0.61055 (5)	0.14183 (2)	0.26873 (4)	0.03384 (16)	
O1	0.5987 (5)	−0.10366 (18)	0.4895 (4)	0.0429 (11)	
O2	0.4138 (5)	0.1473 (2)	0.3258 (4)	0.0426 (11)	
H2A	0.415425	0.132447	0.389072	0.064*	
H2B	0.394335	0.179657	0.338452	0.064*	
N1	0.5897 (6)	0.2176 (2)	0.2310 (4)	0.0372 (13)	
N2	0.7066 (6)	0.1756 (2)	0.4063 (4)	0.0351 (12)	
N3	0.7814 (6)	0.1366 (2)	0.1933 (5)	0.0367 (12)	
N4	0.5283 (6)	0.1109 (2)	0.1259 (5)	0.0395 (13)	
N5	0.6388 (6)	0.0670 (2)	0.3238 (4)	0.0342 (12)	
N6	0.8211 (7)	−0.1061 (2)	0.4606 (5)	0.0465 (15)	
H6A	0.834866	−0.136811	0.487829	0.056*	
H6B	0.889046	−0.089571	0.436429	0.056*	
C1	0.5144 (8)	0.2376 (3)	0.1440 (6)	0.0437 (17)	
H1	0.468971	0.215078	0.092085	0.052*	
C2	0.5013 (8)	0.2896 (3)	0.1277 (7)	0.053 (2)	
H2	0.446150	0.302307	0.066209	0.063*	
C3	0.5683 (8)	0.3228 (3)	0.2009 (7)	0.053 (2)	
H3	0.561552	0.358626	0.189826	0.064*	
C4	0.6444 (8)	0.3036 (3)	0.2896 (6)	0.0463 (18)	
H4	0.691237	0.325956	0.341113	0.056*	
C5	0.6532 (7)	0.2510 (3)	0.3044 (6)	0.0391 (15)	
C6	0.7253 (7)	0.2268 (3)	0.3995 (6)	0.0378 (15)	
C7	0.8054 (8)	0.2532 (3)	0.4799 (6)	0.0474 (18)	
H7	0.818396	0.288929	0.473048	0.057*	
C8	0.8655 (8)	0.2284 (3)	0.5686 (6)	0.051 (2)	
H8	0.922138	0.246229	0.622999	0.061*	
C9	0.8419 (8)	0.1766 (3)	0.5773 (6)	0.0476 (18)	
H9	0.880168	0.158475	0.638995	0.057*	
C10	0.7622 (7)	0.1516 (3)	0.4956 (5)	0.0376 (15)	
H10	0.745896	0.116144	0.502737	0.045*	
C11	0.9100 (7)	0.1537 (3)	0.2309 (7)	0.0427 (17)	
H11	0.925296	0.167870	0.300561	0.051*	
C12	1.0178 (8)	0.1511 (3)	0.1719 (7)	0.0488 (19)	
H12	1.106061	0.163383	0.200527	0.059*	
C13	0.9978 (8)	0.1306 (3)	0.0709 (7)	0.0491 (19)	
H13	1.071896	0.128522	0.029288	0.059*	
C14	0.8690 (8)	0.1133 (3)	0.0312 (6)	0.0438 (17)	
H14	0.853294	0.099132	−0.038411	0.053*	
C15	0.7616 (7)	0.1166 (2)	0.0934 (6)	0.0376 (15)	
C16	0.6201 (8)	0.1007 (3)	0.0561 (6)	0.0394 (16)	
C17	0.5790 (9)	0.0771 (3)	−0.0413 (6)	0.0484 (18)	
H17	0.644015	0.070493	−0.089433	0.058*	
C18	0.4433 (9)	0.0636 (3)	−0.0673 (6)	0.055 (2)	
H18	0.413769	0.046924	−0.132983	0.066*	
C19	0.3503 (9)	0.0744 (3)	0.0034 (6)	0.0521 (19)	
H19	0.255877	0.065725	−0.013631	0.063*	
C20	0.3956 (8)	0.0979 (3)	0.0983 (6)	0.0450 (17)	
H20	0.330940	0.105272	0.146332	0.054*	
C21	0.7662 (7)	0.0492 (3)	0.3579 (6)	0.0402 (16)	
H21	0.842514	0.071019	0.352294	0.048*	
C22	0.7920 (7)	0.0012 (3)	0.4005 (6)	0.0392 (16)	
H22	0.883545	−0.009265	0.424810	0.047*	
C23	0.6833 (7)	−0.0314 (3)	0.4073 (5)	0.0378 (15)	
C24	0.5510 (7)	−0.0144 (3)	0.3708 (6)	0.0401 (16)	
H24	0.474028	−0.036110	0.374114	0.048*	
C25	0.5324 (7)	0.0347 (3)	0.3295 (5)	0.0372 (15)	
H25	0.441787	0.045920	0.304487	0.045*	
C26	0.6994 (8)	−0.0841 (3)	0.4557 (6)	0.0398 (16)	
S1	0.2217 (2)	0.25487 (8)	0.41915 (16)	0.0494 (5)	
F1	0.2188 (6)	0.3402 (2)	0.3117 (4)	0.0755 (15)	
F2	0.1589 (6)	0.2750 (2)	0.2159 (4)	0.0760 (16)	
F3	0.0165 (5)	0.3069 (2)	0.3194 (4)	0.0780 (16)	
O5	0.3606 (6)	0.2454 (2)	0.3927 (5)	0.0561 (14)	
O6	0.1339 (6)	0.2106 (2)	0.4103 (5)	0.0621 (15)	
O7	0.2149 (6)	0.2865 (2)	0.5113 (4)	0.0576 (15)	
C53	0.1482 (10)	0.2957 (3)	0.3155 (8)	0.065 (3)	
S2A	0.1522 (2)	−0.03696 (10)	0.29352 (18)	0.0434 (5)	0.85
F5	−0.0471 (6)	0.0206 (2)	0.2038 (4)	0.0841 (19)	
F4	0.1073 (7)	0.0024 (2)	0.1044 (4)	0.0848 (19)	
O8	0.0628 (5)	−0.0594 (2)	0.3648 (4)	0.0503 (13)	
O4	0.2389 (6)	−0.0727 (2)	0.2487 (5)	0.0636 (16)	
C27A	0.0347 (11)	−0.0160 (4)	0.1798 (8)	0.050 (2)	0.85
F6A	−0.0419 (6)	−0.0556 (3)	0.1393 (5)	0.0708 (18)	0.85
O3A	0.2141 (8)	0.0121 (3)	0.3310 (6)	0.068 (2)	0.85
S2B	0.0994 (15)	−0.0604 (5)	0.2581 (11)	0.043 (3)	0.15
O3B	0.015 (7)	−0.080 (3)	0.185 (5)	0.107 (19)	0.15
C27B	0.110 (5)	0.0032 (10)	0.230 (4)	0.043 (11)	0.15
F6B	0.131 (9)	0.033 (3)	0.283 (6)	0.17 (3)	0.15

### Aquabis(2,2'-bipyridine-κ2N,N')(isonicotinamide-κN)ruthenium(II) bis(trifluoromethanesulfonate) Atomic displacement parameters (Å^2^)

**Table:**

	*U*^11^	*U*^22^	*U*^33^	*U*^12^	*U*^13^	*U*^23^
Ru1	0.0356 (3)	0.0340 (3)	0.0319 (3)	−0.0025 (2)	0.0042 (2)	0.0010 (2)
O1	0.050 (3)	0.033 (3)	0.047 (3)	−0.006 (2)	0.012 (2)	0.003 (2)
O2	0.044 (3)	0.049 (3)	0.037 (3)	−0.004 (2)	0.010 (2)	0.000 (2)
N1	0.037 (3)	0.041 (3)	0.035 (3)	0.000 (2)	0.008 (2)	0.006 (2)
N2	0.038 (3)	0.032 (3)	0.035 (3)	0.001 (2)	0.004 (2)	0.001 (2)
N3	0.041 (3)	0.028 (3)	0.042 (3)	0.000 (2)	0.008 (2)	0.008 (2)
N4	0.044 (3)	0.037 (3)	0.038 (3)	0.004 (3)	0.006 (3)	0.004 (3)
N5	0.038 (3)	0.032 (3)	0.033 (3)	−0.002 (2)	0.005 (2)	0.001 (2)
N6	0.052 (4)	0.032 (3)	0.057 (4)	−0.001 (3)	0.014 (3)	0.005 (3)
C1	0.048 (4)	0.041 (4)	0.041 (4)	0.003 (3)	0.002 (3)	0.008 (3)
C2	0.050 (4)	0.052 (5)	0.054 (5)	0.005 (4)	−0.001 (4)	0.023 (4)
C3	0.056 (5)	0.037 (4)	0.067 (5)	−0.003 (4)	0.004 (4)	0.011 (4)
C4	0.051 (4)	0.035 (4)	0.052 (4)	−0.003 (3)	0.004 (4)	0.006 (3)
C5	0.037 (4)	0.040 (4)	0.042 (4)	−0.004 (3)	0.007 (3)	0.000 (3)
C6	0.040 (4)	0.034 (4)	0.041 (4)	0.001 (3)	0.008 (3)	0.003 (3)
C7	0.050 (4)	0.033 (4)	0.058 (5)	−0.005 (3)	0.001 (4)	−0.010 (3)
C8	0.051 (4)	0.047 (5)	0.051 (5)	0.000 (4)	−0.008 (4)	−0.017 (4)
C9	0.050 (4)	0.049 (5)	0.040 (4)	0.009 (4)	−0.005 (3)	−0.007 (3)
C10	0.044 (4)	0.031 (3)	0.038 (4)	0.002 (3)	0.003 (3)	0.000 (3)
C11	0.042 (4)	0.029 (3)	0.058 (5)	−0.003 (3)	0.009 (3)	0.003 (3)
C12	0.041 (4)	0.036 (4)	0.072 (5)	−0.002 (3)	0.015 (4)	0.004 (4)
C13	0.052 (5)	0.033 (4)	0.067 (5)	0.007 (3)	0.027 (4)	0.007 (4)
C14	0.053 (4)	0.035 (4)	0.045 (4)	0.007 (3)	0.016 (3)	0.008 (3)
C15	0.047 (4)	0.023 (3)	0.044 (4)	0.007 (3)	0.011 (3)	0.004 (3)
C16	0.052 (4)	0.029 (3)	0.038 (4)	0.009 (3)	0.006 (3)	0.005 (3)
C17	0.062 (5)	0.043 (4)	0.040 (4)	0.008 (4)	0.007 (4)	0.000 (3)
C18	0.070 (6)	0.057 (5)	0.036 (4)	0.000 (4)	−0.006 (4)	−0.006 (4)
C19	0.048 (4)	0.057 (5)	0.049 (5)	0.001 (4)	−0.001 (4)	−0.003 (4)
C20	0.042 (4)	0.050 (4)	0.042 (4)	−0.001 (3)	0.002 (3)	−0.003 (3)
C21	0.041 (4)	0.037 (4)	0.044 (4)	−0.004 (3)	0.010 (3)	−0.003 (3)
C22	0.037 (4)	0.032 (4)	0.049 (4)	−0.002 (3)	0.007 (3)	−0.002 (3)
C23	0.047 (4)	0.032 (4)	0.035 (4)	−0.003 (3)	0.011 (3)	0.000 (3)
C24	0.042 (4)	0.041 (4)	0.039 (4)	−0.008 (3)	0.010 (3)	−0.001 (3)
C25	0.038 (4)	0.037 (4)	0.036 (4)	−0.005 (3)	0.001 (3)	−0.003 (3)
C26	0.048 (4)	0.033 (4)	0.038 (4)	−0.003 (3)	0.009 (3)	−0.006 (3)
S1	0.0465 (10)	0.0490 (11)	0.0523 (11)	0.0038 (8)	0.0047 (8)	−0.0023 (9)
F1	0.090 (4)	0.065 (3)	0.068 (3)	−0.002 (3)	−0.002 (3)	0.013 (3)
F2	0.076 (4)	0.102 (4)	0.047 (3)	0.011 (3)	−0.001 (3)	−0.011 (3)
F3	0.059 (3)	0.104 (5)	0.068 (3)	0.017 (3)	−0.002 (3)	0.007 (3)
O5	0.048 (3)	0.058 (4)	0.064 (4)	0.004 (3)	0.012 (3)	−0.005 (3)
O6	0.057 (3)	0.051 (3)	0.079 (4)	−0.011 (3)	0.011 (3)	−0.005 (3)
O7	0.061 (3)	0.062 (4)	0.049 (3)	0.011 (3)	0.006 (3)	−0.012 (3)
C53	0.067 (6)	0.043 (5)	0.076 (6)	0.008 (4)	−0.026 (5)	−0.015 (4)
S2A	0.0361 (11)	0.0552 (14)	0.0382 (11)	0.0036 (11)	0.0025 (9)	0.0043 (11)
F5	0.097 (4)	0.089 (4)	0.073 (4)	0.056 (3)	0.034 (3)	0.036 (3)
F4	0.106 (4)	0.089 (4)	0.068 (3)	0.036 (4)	0.045 (3)	0.034 (3)
O8	0.047 (3)	0.057 (3)	0.049 (3)	0.013 (3)	0.011 (2)	0.018 (3)
O4	0.057 (3)	0.076 (4)	0.060 (4)	0.029 (3)	0.018 (3)	0.012 (3)
C27A	0.058 (6)	0.046 (5)	0.048 (5)	0.016 (5)	0.011 (5)	0.011 (4)
F6A	0.054 (4)	0.091 (5)	0.062 (4)	−0.010 (3)	−0.012 (3)	−0.011 (4)
O3A	0.058 (4)	0.087 (6)	0.060 (4)	−0.028 (4)	0.007 (4)	−0.027 (4)
S2B	0.047 (6)	0.034 (5)	0.049 (6)	−0.001 (5)	0.009 (5)	0.000 (5)
O3B	0.11 (3)	0.11 (3)	0.10 (2)	−0.006 (18)	0.014 (18)	0.021 (18)
C27B	0.044 (14)	0.043 (14)	0.041 (14)	−0.002 (10)	0.007 (10)	0.000 (9)
F6B	0.18 (3)	0.15 (3)	0.17 (3)	0.00 (2)	0.02 (2)	0.009 (19)

### Aquabis(2,2'-bipyridine-κ2N,N')(isonicotinamide-κN)ruthenium(II) bis(trifluoromethanesulfonate) Geometric parameters (Å, º)

**Table:**

Ru1—N3	2.030 (6)	C13—C14	1.374 (11)
Ru1—N1	2.047 (6)	C13—H13	0.9500
Ru1—N4	2.050 (6)	C14—C15	1.392 (10)
Ru1—N2	2.069 (5)	C14—H14	0.9500
Ru1—N5	2.087 (5)	C15—C16	1.467 (10)
Ru1—O2	2.145 (5)	C16—C17	1.392 (10)
O1—C26	1.234 (8)	C17—C18	1.374 (12)
O2—H2A	0.8881	C17—H17	0.9500
O2—H2B	0.8881	C18—C19	1.383 (12)
N1—C1	1.351 (9)	C18—H18	0.9500
N1—C5	1.367 (9)	C19—C20	1.372 (11)
N2—C10	1.347 (8)	C19—H19	0.9500
N2—C6	1.358 (9)	C20—H20	0.9500
N3—C15	1.359 (9)	C21—C22	1.381 (10)
N3—C11	1.364 (9)	C21—H21	0.9500
N4—C20	1.346 (9)	C22—C23	1.375 (9)
N4—C16	1.363 (9)	C22—H22	0.9500
N5—C21	1.351 (9)	C23—C24	1.392 (10)
N5—C25	1.351 (8)	C23—C26	1.511 (10)
N6—C26	1.317 (9)	C24—C25	1.393 (10)
N6—H6A	0.8799	C24—H24	0.9500
N6—H6B	0.8799	C25—H25	0.9500
C1—C2	1.382 (11)	S1—O7	1.438 (6)
C1—H1	0.9500	S1—O6	1.441 (6)
C2—C3	1.377 (12)	S1—O5	1.462 (6)
C2—H2	0.9500	S1—C53	1.773 (9)
C3—C4	1.363 (11)	F1—C53	1.361 (10)
C3—H3	0.9500	F2—C53	1.387 (11)
C4—C5	1.392 (10)	F3—C53	1.329 (11)
C4—H4	0.9500	S2A—O4	1.428 (6)
C5—C6	1.461 (10)	S2A—O8	1.457 (6)
C6—C7	1.389 (10)	S2A—O3A	1.474 (8)
C7—C8	1.365 (11)	S2A—C27A	1.810 (10)
C7—H7	0.9500	F5—C27A	1.308 (10)
C8—C9	1.383 (11)	F5—C27B	1.60 (5)
C8—H8	0.9500	F4—C27A	1.349 (11)
C9—C10	1.379 (10)	F4—C27B	1.59 (5)
C9—H9	0.9500	O8—S2B	1.438 (14)
C10—H10	0.9500	O4—S2B	1.423 (14)
C11—C12	1.368 (10)	C27A—F6A	1.345 (12)
C11—H11	0.9500	S2B—O3B	1.27 (7)
C12—C13	1.376 (12)	S2B—C27B	1.71 (2)
C12—H12	0.9500	C27B—F6B	1.03 (8)
			
N3—Ru1—N1	91.1 (2)	C13—C14—C15	119.7 (7)
N3—Ru1—N4	79.4 (2)	C13—C14—H14	120.1
N1—Ru1—N4	99.3 (2)	C15—C14—H14	120.1
N3—Ru1—N2	96.3 (2)	N3—C15—C14	121.3 (7)
N1—Ru1—N2	78.6 (2)	N3—C15—C16	115.3 (6)
N4—Ru1—N2	175.2 (2)	C14—C15—C16	123.4 (7)
N3—Ru1—N5	90.7 (2)	N4—C16—C17	121.3 (7)
N1—Ru1—N5	173.9 (2)	N4—C16—C15	114.1 (6)
N4—Ru1—N5	86.8 (2)	C17—C16—C15	124.6 (7)
N2—Ru1—N5	95.4 (2)	C18—C17—C16	119.4 (8)
N3—Ru1—O2	171.7 (2)	C18—C17—H17	120.3
N1—Ru1—O2	86.9 (2)	C16—C17—H17	120.3
N4—Ru1—O2	93.0 (2)	C17—C18—C19	119.1 (7)
N2—Ru1—O2	91.2 (2)	C17—C18—H18	120.4
N5—Ru1—O2	92.1 (2)	C19—C18—H18	120.4
Ru1—O2—H2A	111.0	C20—C19—C18	119.4 (8)
Ru1—O2—H2B	110.2	C20—C19—H19	120.3
H2A—O2—H2B	103.7	C18—C19—H19	120.3
C1—N1—C5	117.4 (6)	N4—C20—C19	122.4 (7)
C1—N1—Ru1	126.4 (5)	N4—C20—H20	118.8
C5—N1—Ru1	116.1 (4)	C19—C20—H20	118.8
C10—N2—C6	118.0 (6)	N5—C21—C22	123.8 (7)
C10—N2—Ru1	126.8 (5)	N5—C21—H21	118.1
C6—N2—Ru1	114.8 (4)	C22—C21—H21	118.1
C15—N3—C11	117.9 (6)	C23—C22—C21	119.0 (7)
C15—N3—Ru1	115.6 (5)	C23—C22—H22	120.5
C11—N3—Ru1	126.4 (5)	C21—C22—H22	120.5
C20—N4—C16	118.4 (6)	C22—C23—C24	118.4 (6)
C20—N4—Ru1	126.0 (5)	C22—C23—C26	123.3 (7)
C16—N4—Ru1	115.5 (5)	C24—C23—C26	118.2 (6)
C21—N5—C25	117.0 (6)	C23—C24—C25	119.5 (6)
C21—N5—Ru1	120.6 (5)	C23—C24—H24	120.2
C25—N5—Ru1	122.4 (5)	C25—C24—H24	120.2
C26—N6—H6A	120.8	N5—C25—C24	122.2 (6)
C26—N6—H6B	119.1	N5—C25—H25	118.9
H6A—N6—H6B	120.0	C24—C25—H25	118.9
N1—C1—C2	122.3 (7)	O1—C26—N6	123.9 (7)
N1—C1—H1	118.8	O1—C26—C23	118.2 (6)
C2—C1—H1	118.8	N6—C26—C23	117.9 (6)
C3—C2—C1	119.8 (7)	O7—S1—O6	116.1 (4)
C3—C2—H2	120.1	O7—S1—O5	115.0 (4)
C1—C2—H2	120.1	O6—S1—O5	114.1 (4)
C4—C3—C2	119.0 (7)	O7—S1—C53	101.3 (4)
C4—C3—H3	120.5	O6—S1—C53	104.3 (4)
C2—C3—H3	120.5	O5—S1—C53	103.7 (4)
C3—C4—C5	119.6 (7)	F3—C53—F1	108.2 (7)
C3—C4—H4	120.2	F3—C53—F2	107.7 (7)
C5—C4—H4	120.2	F1—C53—F2	102.0 (8)
N1—C5—C4	121.9 (7)	F3—C53—S1	114.3 (8)
N1—C5—C6	114.3 (6)	F1—C53—S1	112.5 (6)
C4—C5—C6	123.8 (7)	F2—C53—S1	111.4 (6)
N2—C6—C7	121.0 (7)	O4—S2A—O8	114.5 (4)
N2—C6—C5	115.2 (6)	O4—S2A—O3A	117.3 (4)
C7—C6—C5	123.7 (7)	O8—S2A—O3A	114.1 (4)
C8—C7—C6	120.6 (7)	O4—S2A—C27A	103.5 (4)
C8—C7—H7	119.7	O8—S2A—C27A	104.0 (4)
C6—C7—H7	119.7	O3A—S2A—C27A	100.7 (5)
C7—C8—C9	118.3 (7)	F5—C27A—F6A	109.1 (9)
C7—C8—H8	120.9	F5—C27A—F4	107.1 (7)
C9—C8—H8	120.9	F6A—C27A—F4	108.9 (8)
C10—C9—C8	119.4 (7)	F5—C27A—S2A	112.7 (7)
C10—C9—H9	120.3	F6A—C27A—S2A	109.6 (6)
C8—C9—H9	120.3	F4—C27A—S2A	109.4 (7)
N2—C10—C9	122.5 (7)	O3B—S2B—O4	113 (3)
N2—C10—H10	118.7	O3B—S2B—O8	118 (3)
C9—C10—H10	118.7	O4—S2B—O8	116.0 (11)
N3—C11—C12	122.3 (7)	O3B—S2B—C27B	108 (4)
N3—C11—H11	118.8	O4—S2B—C27B	96.7 (19)
C12—C11—H11	118.8	O8—S2B—C27B	101.7 (19)
C11—C12—C13	119.7 (8)	F6B—C27B—F5	91 (6)
C11—C12—H12	120.1	F6B—C27B—F4	129 (5)
C13—C12—H12	120.1	F5—C27B—F4	84 (3)
C14—C13—C12	119.0 (7)	F6B—C27B—S2B	129 (6)
C14—C13—H13	120.5	F5—C27B—S2B	104 (2)
C12—C13—H13	120.5	F4—C27B—S2B	101 (2)
			
C5—N1—C1—C2	0.3 (11)	C16—C17—C18—C19	1.1 (12)
Ru1—N1—C1—C2	176.9 (6)	C17—C18—C19—C20	−0.8 (13)
N1—C1—C2—C3	1.2 (12)	C16—N4—C20—C19	0.6 (11)
C1—C2—C3—C4	−1.4 (13)	Ru1—N4—C20—C19	−175.8 (6)
C2—C3—C4—C5	0.1 (12)	C18—C19—C20—N4	−0.1 (13)
C1—N1—C5—C4	−1.6 (10)	C25—N5—C21—C22	−2.1 (10)
Ru1—N1—C5—C4	−178.5 (6)	Ru1—N5—C21—C22	176.5 (5)
C1—N1—C5—C6	176.3 (6)	N5—C21—C22—C23	1.4 (11)
Ru1—N1—C5—C6	−0.6 (8)	C21—C22—C23—C24	−0.1 (10)
C3—C4—C5—N1	1.4 (12)	C21—C22—C23—C26	−178.0 (6)
C3—C4—C5—C6	−176.3 (7)	C22—C23—C24—C25	−0.4 (10)
C10—N2—C6—C7	3.3 (10)	C26—C23—C24—C25	177.6 (6)
Ru1—N2—C6—C7	−170.7 (6)	C21—N5—C25—C24	1.5 (10)
C10—N2—C6—C5	−175.3 (6)	Ru1—N5—C25—C24	−177.0 (5)
Ru1—N2—C6—C5	10.7 (8)	C23—C24—C25—N5	−0.3 (10)
N1—C5—C6—N2	−6.7 (9)	C22—C23—C26—O1	157.0 (7)
C4—C5—C6—N2	171.2 (7)	C24—C23—C26—O1	−20.9 (10)
N1—C5—C6—C7	174.8 (7)	C22—C23—C26—N6	−22.0 (10)
C4—C5—C6—C7	−7.4 (11)	C24—C23—C26—N6	160.1 (7)
N2—C6—C7—C8	−1.1 (12)	O7—S1—C53—F3	−64.7 (7)
C5—C6—C7—C8	177.4 (7)	O6—S1—C53—F3	56.1 (7)
C6—C7—C8—C9	−1.5 (12)	O5—S1—C53—F3	175.8 (6)
C7—C8—C9—C10	1.7 (12)	O7—S1—C53—F1	59.1 (8)
C6—N2—C10—C9	−3.1 (10)	O6—S1—C53—F1	179.9 (7)
Ru1—N2—C10—C9	170.1 (5)	O5—S1—C53—F1	−60.4 (8)
C8—C9—C10—N2	0.6 (12)	O7—S1—C53—F2	172.9 (6)
C15—N3—C11—C12	−0.1 (10)	O6—S1—C53—F2	−66.3 (8)
Ru1—N3—C11—C12	−176.3 (5)	O5—S1—C53—F2	53.4 (7)
N3—C11—C12—C13	−0.1 (11)	O4—S2A—C27A—F5	−174.7 (7)
C11—C12—C13—C14	0.2 (11)	O8—S2A—C27A—F5	65.3 (8)
C12—C13—C14—C15	−0.1 (11)	O3A—S2A—C27A—F5	−53.0 (8)
C11—N3—C15—C14	0.2 (9)	O4—S2A—C27A—F6A	63.6 (7)
Ru1—N3—C15—C14	176.8 (5)	O8—S2A—C27A—F6A	−56.3 (7)
C11—N3—C15—C16	−177.9 (6)	O3A—S2A—C27A—F6A	−174.7 (6)
Ru1—N3—C15—C16	−1.3 (7)	O4—S2A—C27A—F4	−55.7 (8)
C13—C14—C15—N3	−0.1 (10)	O8—S2A—C27A—F4	−175.7 (6)
C13—C14—C15—C16	177.8 (6)	O3A—S2A—C27A—F4	66.0 (7)
C20—N4—C16—C17	−0.3 (10)	O3B—S2B—C27B—F6B	150 (9)
Ru1—N4—C16—C17	176.5 (5)	O4—S2B—C27B—F6B	−93 (9)
C20—N4—C16—C15	179.6 (6)	O8—S2B—C27B—F6B	25 (9)
Ru1—N4—C16—C15	−3.5 (7)	O3B—S2B—C27B—F5	47 (4)
N3—C15—C16—N4	3.2 (8)	O4—S2B—C27B—F5	164 (2)
C14—C15—C16—N4	−174.9 (6)	O8—S2B—C27B—F5	−78 (3)
N3—C15—C16—C17	−176.9 (6)	O3B—S2B—C27B—F4	−39 (4)
C14—C15—C16—C17	5.1 (11)	O4—S2B—C27B—F4	77 (3)
N4—C16—C17—C18	−0.5 (11)	O8—S2B—C27B—F4	−164 (2)
C15—C16—C17—C18	179.5 (7)		

### Aquabis(2,2'-bipyridine-κ2N,N')(isonicotinamide-κN)ruthenium(II) bis(trifluoromethanesulfonate) Hydrogen-bond geometry (Å, º)

**Table:**

*D*—H···*A*	*D*—H	H···*A*	*D*···*A*	*D*—H···*A*
O2—H2*A*···O1^i^	0.89	1.73	2.617 (7)	174
O2—H2*B*···O5	0.89	1.90	2.775 (8)	169
N6—H6*A*···O6^i^	0.88	2.32	3.190 (9)	169

Symmetry code: (i) −*x*+1, −*y*, −*z*+1.
